# New Perspectives in Food Allergy

**DOI:** 10.3390/ijms21041474

**Published:** 2020-02-21

**Authors:** Massimo De Martinis, Maria Maddalena Sirufo, Mariano Suppa, Lia Ginaldi

**Affiliations:** 1 Department of Life, Health and Environmental Sciences, University of L’Aquila, 67100 L’Aquila, Italy; maddalena.sirufo@gmail.com (M.M.S.); lia.ginaldi@cc.univaq.it (L.G.); 2 Allergy and Clinical Immunology Unit, Center for the diagnosis and treatment of Osteoporosis, AUSL 04 Teramo, Italy; 3 Department of Dermatology, Hôpital Erasme, Université Libre de Bruxelles, 1070 Brussels, Belgium; dr.marianosuppa@gmail.com

**Keywords:** food allergy, immune tolerance, allergic sensitization, gut microbiota, mucosal immunity, food allergy treatment, translational immunology

## Abstract

The improvement of the knowledge of the pathophysiological mechanisms underlying the tolerance and sensitization to food antigens has recently led to a radical change in the clinical approach to food allergies. Epidemiological studies show a global increase in the prevalence of food allergy all over the world and manifestations of food allergy appear increasingly frequent also in elderly subjects. Environmental and nutritional changes have partly changed the epidemiology of allergic reactions to foods and new food allergic syndromes have emerged in recent years. The deepening of the study of the intestinal microbiota has highlighted important mechanisms of immunological adaptation of the mucosal immune system to food antigens, leading to a revolution in the concept of immunological tolerance. As a consequence, new prevention models and innovative therapeutic strategies aimed at a personalized approach to the patient affected by food allergy are emerging. This review focuses on these new perspectives and their practical implications in the management of food allergy, providing an updated view of this complex pathology.

## 1. Introduction

Food allergy (FA) is an adverse reaction to a specific food antigen, normally harmless to the healthy population, which is mediated by immunological mechanisms and arises in an individual susceptible to that specific allergen [1,2].

FA, therefore, differs from adverse reactions caused by toxins or pathogens contained in the food, as well as from the so-called food intolerances, which exhibit the same symptoms but recognize different pathogenetic mechanisms. Intolerances are defined as nonimmune reactions, mediated by toxic, pharmacological, metabolic, and undefined mechanisms. Milk intolerance due to the deficiency of the enzyme lactase, normally present in the brush border of the intestinal mucosa, and adverse reactions to foods characterized by a high histamine content or histamine-liberating substances, such as strawberries, chocolate, alcoholic drinks, and fermented cheeses, are examples of nonimmune mediated food intolerances [3]. In the past, FAs and intolerances were often confused with each other, due to their clinical similarity. Moreover, the same food is often responsible for both intolerance and allergy, rendering diagnosis difficult.

What distinguishes FA from other adverse reactions to foods is, therefore, the underlying pathogenetic mechanism: FAis an adverse reaction arising from a specific immune response that occurs reproducibly on exposure to a certain food. Moreover, based on the specific immunopathogenetic mechanism, it is possible to distinguish immunoglobulin E IgE-mediated FAs from non-IgE-mediated and mixed reactions to foods [4].

In this review, we specifically deal with IgE-mediated FAs, as major advances in knowledge of pathogenic mechanisms, as well as in diagnosis and therapy, have recently been made especially in this field.

## 2. Epidemiology

FA is very common worldwide and is becoming a major public health problem. Although precise epidemiological data are lacking, it is clear that the prevalence of FA has increased significantly in the last two decades in Western countries, where rates of up to 10% have been documented among preschool children [5,6]. It is estimated that over 220 million people worldwide suffer from FA [3,7,8,9,10]. However, making precise estimates is not easy due to the multiplicity and variable severity of clinical presentations, the difficulty in making objective diagnoses because of the strong psychological influences on the subjective perception of disease, and finally the complexity of the diagnostic tools. More than one-third of parents report food hypersensitivity reactions in their children, but the prevalence of objectively diagnosed and verifiable FA in the first year of life actually varies from 6% to 10% and drops to 2% to 5% in adulthood.

FA mainly affects children [11] but an increasing number of elderly subjects also have symptoms of FA [12]. Age-related gender differences are reported in FA [13]. For a long time, FA has been considered an almost exclusively pediatric disease because in the majority of cases it begins in childhood and tends to disappear with growth. However, the current exponential growth of the adult and elderly population, especially in Western countries, and the environmental and lifestyle changes, have profoundly changed the epidemiology of FA with a growing increase even in advanced age. Moreover, FA in aging exhibits peculiar clinical features and immunopathogenetic mechanisms, increasing the diagnostic complexity [14].

The exponential growth of FA and the significant variability of the geographical distribution are the result of the influence of environmental factors and lifestyles on genetic predisposition [15,16]. The highest prevalence of FA has been observed in Australia, reaching 10% of the infant population [17,18].

FA includes a wide spectrum of clinical manifestations, from mild forms with organ localization, to serious and potentially fatal forms with systemic involvement. Almost half of patients with IgE-dependent FA have experienced at least one serious anaphylactic reaction, especially in childhood and adolescence [11,19]. The variability of clinical expressions and the complexity of the underlying immunological mechanisms contribute to making diagnosis often difficult and complicate the studies on the epidemiology of FA [20,21,22,23,24,25,26,27,28,29,30].

Although each type of food may constitute a potential allergen, the list of foods responsible for the great majority of cases, especially the most clinically severe forms, is relatively short [3,10,24]. In industrialized countries the foods most frequently responsible for allergies in children are cow’smilk, eggs, wheat, fish and shellfish, peanuts, walnuts, and soybeans. However, in 80% of cases, children with IgE-non-dependent FA recover within three years of life [31]. Fish and seafood, peanuts and nuts, and fruit and vegetables are the prevalent causal allergens in adults. Furthermore, in relation to the eating habits, different foods may also be responsible for allergic sensitization in other countries. For example, school-age Mexican children more often exhibit hypersensitivity reactions to chocolate, strawberries, crustaceans, and eggs [32], while sensitization to nuts and peanuts is more common in Chile [33], to chili, walnuts, chocolate, milk, and prawnsin El Salvador [34], and to fruits, vegetables, and seafood in Colombia [35,36].

A growing prevalence of FA has been recently shown in developing countries. Incidence rates similar to those of Western countries have been reported in China and Africa. The economic growth of countries such as China and the expansion of the phenomenon of globalization point to a future increase of FA prevalence. Interestingly, children of Asian or African origin born in the West are at greater risk of developing FA than Caucasian children, suggesting the importance of the genome–environment–lifestyle interaction in the determinism of this pathology. Interestingly, Panjari et al. showed that among children of Asian origin born in Australia the prevalence of allergy to nuts is double, while Asian children who immigrated to Australia at 5 years of age are protected [37], confirming epigenetic and environmental impacts on disease development. Such epidemiological studies on the geographical distribution and prevalence of FA are essential for a better understanding of its pathogenesis and above all for the planning of possible preventive interventions [38]. For example, the increase in FA observed in Australia has been linked to effective anti-skin cancer campaigns, which, while on the one hand, have decreased melanoma cases through a decreased exposure to ultraviolet rays, on the other, they have led to an increase in FA and osteoporosis due to the resulting high rates of vitamin D deficiency, which is known to counteract both diseases. In this case, prevention programs based on correct dietary supplementation with vitamin D could be useful for both FA and osteoporosis [39,40,41].

## 3. Clinical Presentation and Natural History

The clinical manifestations of FA are highly variable and the natural history can change from patient to patient, as well as the expression of biomarkers and the response to various therapeutic approaches. Understanding the natural history of FA is essential to successfully plan patients’management. The natural history of FA is different in children and adults [4]. Most FAs begin within the first two years of life, but the type of the causative allergens affect the natural history, as well as the risk of systemic reactions [1]. Allergic reactions to cow’s milk and eggs usually disappears with age, while allergy to peanuts, nuts, and seafoods more often persists even into adulthood; moreover, around 30% of patients develop more than one type of allergic sensitization over time [42]. Fruit sensitization appears belatedly, exhibiting various cross-reactivities, which give rise to peculiar clinical manifestations, such as pollen–food allergy syndrome [43,44].

Clinical manifestations of FA can affect various organs and systems, including skin, gut and respiratory, and cardiovascular and nervous systems; moreover, symptoms variously embrace eachother, giving rise to complex disease pictures. Interestingly, in patients affected by FA, the skin may be one of the most common target organs, with clinical manifestations including pruritus and urticaria, but also an important site of primary sensitization to food allergens, so that atopic dermatitisisconsidered a risk factor for the development of FA [45]. Recently, a clinical-pathogenetic classification has been proposed, in which different phenotypes (clinical presentation) and endotypes (underlying pathobiological mechanism) are recognized, that share a fair degree of overlap [46]. Clinical expressions are extremely variable, ranging from mild and localized manifestations of hypersensitivity to foods, such as oral itching, to serious, systemic, and often fatal reactions, such as anaphylactic shock [20].

Environmental factors can favor the triggering of a FA and the severity of its clinical manifestation [47]. Several facilitating factors could trigger allergic reactions following the ingestion of a food allergen, including exercise, alcohol, and drugs, such as antacids and non-steroidal anti-inflammatory drugs (NSAIDs), menses, and infections.For example, urticaria or anaphylaxis can be triggered following physical exercise performed by the patient within 2 to 4 h from the ingestion of the culprit foods (food-dependent exercise-induced anaphylaxis (FDEIA)). In this particular allergic phenotype, which mainly affects the female sex and is frequent in adolescents and young adults, the food responsible is mainly wheat, although milk, soy, celery, and seafood are also implicated.

It has been suggested that physical exercise, as well as the other facilitating factors, both increases the gastrointestinal absorption of food allergens and lowers the threshold for mastcell and basophil degranulation in sensitized individuals [48,49]. The great majority of patients with wheat-dependent exercise-induced anaphylaxis (WDEIA) have serum IgE specific for omega-5 gliadin and only in a minority of cases for high molecular weight glutenin (HMW-glutenin) [50].

The alpha-galsyndromeisan unusualform of IgE-mediated in which the sensitizing allergenis galactose-alpha-1,3-galactose contained in the tissues of most mammals. The reaction to ingestion of red meat (anaphylaxis, hypotension, urticaria, angioedema) occurs late (from 3 to 6 h) compared to the classic immediate reactions [51,52,53]. This syndrome is mainly described in the United States, Australia, and Germany but also in increasing numbers in other countries, and sensitization is thought to arise following a tickbite, mostly “Amblyomma americanum” or “Lone star”. Alpha-gal IgE positive patients are at risk of anaphylactic shock following exposure to a biologic used as antiblastic agent in second-line therapy of recurrent colorectal, head, and neck cancers. It is a chimeric mouse-human monoclonal antibody against the epidermal growth factor receptor because of the presence of alpha-gal on the mouse-derived Fab portion of its heavy chain [54,55].

Local or systemic manifestations of FA may arise even following exposure to inhalant allergens as a consequence of structural homology between plant pollen and food allergens or following exposure to aerosolized food proteins (FA with aerosol sensitization) [56]. This latter phenotype is more frequent in patients who work in the food industry (occupational allergies) and is characterized by prevalent respiratory symptoms (asthma and rhinitis), anaphylaxis, and urticaria. The most common causal foods include wheat and other cereals, shellfish, and vegetables [57,58].

The oral allergic syndrome (OAS), more common in adults and adolescents, presents symptoms mainly affecting the oropharynx, and depends on a polysensitization to inhalant and food allergens (pollen–food allergy syndrome). Rawapple, peanuts, almonds, hazelnuts, and other fruits of the Rosaceae family are commonly implicated in patients with allergy to birch, while banana, kiwi, and melon are the eliciting foods in patients allergic to ambrosia, and melon and tomato are responsible for symptoms ingrass allergy patients. In this phenotype, the IgE against pollen cross-reacts with homologous proteins in plant foods [59]. The responsible IgE are initially directed against pollen, which, if inhaled, induces rhinitis. The contact of the rawfruit, for example peach or apple, with the oral mucosa immediately triggers itching, oral burning, and angioedema of the lips, tongue, and palate [60]. These are mostly cross-reactions with thermolabile food epitopes and, consequently, in skin diagnostic tests, it is better to use rawfruit or vegetables (puncture by puncture) [61].

The latex–food allergy syndrome is observed in up to 50% of patients with latex allergy. These patients have hypersensitivity to several vegetable foods, such as banana, kiwi, avocado, tomato, potato, chestnut, peach, and pepper [50,62]. Latex allergy, very common in industrialized countries in the 1990s due to the widespread use of latex gloves and other devices, is now decreasing thanks to the growing use of alternative materials.

## 4. FA Diagnostics

A precise and accurate diagnosis is essential for a personalized management of patients with FA. In this context, more sophisticated and accurate diagnostic tests, including component-resolved diagnostics and the epitope reactivity, can allow more targeted diagnosis, more precise prognostic evaluations, and better choice of the therapeutic approach. Clinical history, prick tests, specific serum IgE searching, elimination diets, and subsequent oral food challenges are important FA diagnostic tools [60]. The oral food challenge (OFC) is an essential test for the diagnosis of FA. It can be performed as either open or single-blind food challenge, or better as double-blind placebo-controlled food challenge (DBPCFC). This latter is considered the diagnostic gold standard of FA [63]. It is, however, a complex test that puts the patient at risk of serious anaphylactic reactions [64]. Other tests useful to predict the risk of serious anaphylactic reactions, such as molecular or component-resolved diagnostics (CRD) and the basophil activation test (BAT), have been developed in recent years and can be utilized to plan personalized treatment based on the patient’s molecular and clinical profile [65].

BAT uses the suspected allergen in vitro to stimulate the patient’s basophils. Various surface markers expressed on human basophils, such as CD63, CD69, and CD203c, have been used to demonstrate basophil activation. Among them, CD203c, together with CD63, is considered to be the most reliable activation marker. The activation-linked molecule CD203c is an ectoenzyme located both on the plasma membrane and in the cytoplasmic compartment of basophils. Cross-linking of the high-affinity IgE receptor Fc epsilon RI (FcεRI) by an allergen or anti-IgE antibody results in a rapid upregulation of intracellular CD203c molecules to the cell surface and is accompanied by mediator release. In contrast to CD63, resting basophils show some degree of constitutive CD203c expression on their plasma membrane, whereas CD63 expression is closely related to basophil degranulation. Similar to CD63, basophil CD203c expression rapidly increases after allergen challenge in sensitized individuals. These molecules are, therefore, useful targets for flow cytometry-based tests to analyze sensitized individuals and patients with type I allergy [66]. In fact, the increase in membrane expression of these markers can be detected by flow cytometry, and the result expressed as basophil fluorescence intensity and percentage of positive cells [67]. This test allows a judgment on the degree of allergen-specific reactivity and on the risk of serious reactions to provocation tests [68].

The CRD defines allergic sensitizationat the molecular level using recombinant or purified allergens. It differs from standard tests because it recognizes the responsible molecular epitopes, i.e., the molecular component of the food allergen against which specific IgEs are directed. CRD is used in clinical practice to improve diagnostic accuracy, discriminate true allergic sensitization from cross-sensitization phenomena, and stratify the clinical risk associated with specific sensitization profiles [69]. Foods, as well as allergenic extracts, contain numerous cross-reactive allergenic components. Skin prick test and radio-allergo-sorbent test (RAST) for specific IgE with standard extracts can only indicate sensitization to a food. The CRD allows instead to determine the individual profile of allergic molecular reactivity and also to formulate a prognostic judgment [70,71]. For example, allergic reactions to fruit and vegetables can depend on primary sensitization or cross-reactivity with inhalant allergens. Usually, labile allergens, such as pathogenesis-related (PR) proteins and profilins, are responsible for cross-reactivity, triggering mild clinical manifestations, such as oral reactions, while allergens resistant to both heat and proteolysis, such as storage and lipid-transfer proteins (LTP), are responsible for systemic reactions [72,73,74,75]. For example, apple contains thermolabile allergenic molecules (epitopes) belonging to the family of PR-10 (Mal d1) and profilins (Mal d4), but also thermostable LTP (Mal d3). LTP Mal d3 sensitization is associated with 7-folds greater risk of anaphylaxis than Mal d 1 (Bet v 1 homologous). Mal d3 sensitization is, therefore, considered a marker of mild reactions to fresh fruit and vegetables caused by cross-reactivity with plant pollen (birch) [59]. Peanut allergy is associated with different clinical pictures, and a molecular approach can, therefore.be very useful. Ara h2 is one of the main peanut proteins associated with serious clinical reactions, while Ara h8 is a homologue of labile birch pollen (Bet v1) not responsible for significant clinical reactions [76,77]. In addition, the CRD also allows us to predict the persistence of a FA. Children with prolonged egg allergy have elevated IgE levels against the sequential epitopes Gald1 and Gald2. These children do not tolerate even cooked egg [78,79,80,81].

## 5. Pathophysiology of FA Phenotypes

The immunological mechanisms underlying both local and systemic manifestations of IgE-mediated FA are type I hypersensitivity responses to specific food allergens. During the phase of allergic sensitization, the first contact with the allergen occurs, which determines an initial immunological response, leading to the breaking of tolerance followed by the production of specific IgEs. The first contact usually takes place orally, but other ways of sensitization are also possible. For example, sensitization can occur through altered skin, as in atopic dermatitis, or by food protein aerosols (inhalation route). In the case of pollen–food allergy syndrome, the patient is sensitized to inhaled pollen allergens that cross-react with food allergens [82]. Once produced, IgE anchors to the high-affinity receptor for their Fc fragment on the membrane of tissue mastcells and circulating basophils, whose cytoplasm contains vasoactive substances and mediators of anaphylaxis, such as histamine [61]. So these cells become sensitized and at a second contact with that allergen they activate and degranulate, releasing the anaphylactic mediators in the tissues and in the blood flow. This is the elicitation phase of the allergic reactions that underlie the various clinical manifestations (early phase reaction) [83]. Following effector cells degranulation, the “de novo” production of other immunological mediators also occurs, including platelet activation factor (PAF), leukotrienes, and cytokines such as interleukin (IL)-4, IL-5, and IL-13, which together contribute to the allergic inflammation.

In addition to such immediate phase, there is also a late phase of the IgE-mediated allergic reaction, different from the delayed cellular hypersensitivity reactions. Several chemotactic mediators released in the tissues during the early phase reaction attract other inflammatory effector cells that activate and chronicize inflammation through the production of further inflammatory mediators [84].

Although the early and late phases of allergic inflammation are closely related, the signaling pathways and mediators involved in each of them are different, corresponding to different anatomo-clinical manifestations. The symptoms of the early phase are essentially functional, acute, and rapidly reversible, while in the late phase they are more slowly reversible. The initial phase mainly involves histamine and PAF released by mastcells and is under the control of regulatory T lymphocytes (Treg). If the allergen enters the bloodstream, additional activation pathways can also be mediated by basophils and neutrophils [85]. Specific inflammatory cytokines, including tumor necrosis factor (TNF)-alpha and Th2 cytokines suchas IL-9, IL-31, and IL-33, underly late tissue inflammation [86,87].

If the allergen is spread systemically, histamine- and PAF-induced symptoms affecting organs other than the gastrointestinal system, including skin (urticaria) and lung (asthma), may also occur. Serotonin or 5-hydroxy-tryptamine and PAF play central roles in acute gastrointestinal manifestations, such as with diarrhea. Systemically distributed allergens react not only with mastcells but also with circulating sensitized basophils, eliciting a severe life-threatening systemic reaction characterized by multiple organ and system involvement, hypotension, and shock [88]. Finally, following repeated exposure to the food allergen, allergic inflammation is perpetuated and mastcells increase in tissues, forming the background of persistent gastrointestinal manifestations [74].

## 6. Tolerance Disruption

Currently, tolerance to food antigensis no longer considered a condition of anergy or lack of response of the immune system, but an antigen-specific recognition of food epitopes. Tolerance to foods is an active process induced by oral exposure to food antigens and also supported by breastfeeding through maternal transfer of immune complexes. It, therefore, develops early in life, involves various immune and nonimmune cells, and can be reprogrammed, for example, by inflammatory stimuli, with the consequent onset of allergic reactions [74].

FA, therefore, arises in some individuals with atopic predisposition in which tolerance for food antigens is not established or is interrupted, due to an altered functioning of the gut immune system [89,90]. Dendritic cells (DCs) play central roles in tolerance determinism because, through the processing and presentation of food antigens, they promote the differentiation of naive T cells into forkhead box P3 (Foxp3) positive Treg cells that produce cytokines suppressing allergic reactions, such as growth factor-beta (TGF -β) and IL-10, through a retinoic acid-dependent mechanism [91]. Chemokine receptor CX3CR1 + macrophages also express IL-10 and induces the expansion of Foxp3+ Treg lymphocytes of the lamina propria of the intestinal mucosa by inhibiting sensitization to specific food allergens.

CX3CR1+ macrophages can capture food antigens directly from the intestinal lumen by insinuating dendritic extensions between epithelial junctions (periscopic mechanism). CD103+ DCs transport the captured antigens to Peyer’s patches and mesenteric lymphnodes and drive the homing of B lymphocytes secreting mucosal IgA in the gut, thus contributing to the maintenance of a specific immune tolerance [92]. IgGs, in particular, specific IgG4, can inhibit the initiation as well as the effector phases of allergic responses, thus also contributing to the acquisition of oral tolerance to food antigens [2].

The disruption of the physiological tolerance to food antigens is mainly triggered by danger signals leading to pro-inflammatory cytokine production from intestinal epithelial cells (IECs) [93]. Danger signals, through pathogen-associated molecular patterns (PAMPs) or damage-associated molecular patterns (DAMPs), activate a series of epithelial pathways that result in the production of inflammatory cytokines, such as IL-25 and IL-31, which impact on the antigen presenting cells (APCs), giving them a functional pro-inflammatory phenotype [22]. Other cytokines, including thymic stromal lymphopoietin (TSLP) and IL-33, reprogram the APCs, transforming them into cells capable of shifting the differentiation of naive lymphocytes towards IL-4 and IL-13, producing Th2 cells rather than Tregs, with consequent allergic sensitization [94,95]. Inflammation, favored by the age-related gut barrier and digestive function impairment, as well as by the chronic inflammation that characterizes senescence, plays a central role in the breakdown of tolerance to food antigens in the elderly [14,96,97].

The Th2 cell priming, favored by mucosal DCs induced by food allergens, leads to the production of IL-4, which in turn drives allergic responses, including the expansion of eosinophils and mastcells in the gut mucosa and the isotypical switch in local B cells towards IgE production, further expanding the Th2 lymphocyte pool. Moreover, tolerogenic Treg lymphocytes are suppressed, reprogrammed, and transformed into pro-allergic IL-4-producing cells [98,99]. Among the other cell types involved in tolerance disruption, innate lymphoid cells of type 2 (ILC2), similar to Th2 lymphocytes but lacking specificity for the antigen, and IL-9-producing cells (Th9 cells), which are mucosal mastcells capable of producing various inflammatory cytokines and capable to suppress the function of Tregcells, have been recently recognized [2,100]. All of them increase the vicious circle which leads, through an autocrine loop, to the increase in the intestinal mastcell pool [20,92,101].

Interestingly, both damaged skin or gut barrier dysfunctions may contribute to FA development. In particular, the normal tolerogenic response to food antigens of the intestinal mucosa can be by-passed through skin exposure to food allergens, thus promoting allergic sensitization [102]. Skin DCs, induced by inflammatory cytokines, promote allergic Th2 immune responses rather than tolerogenic responses to antigens. Th2 cells in the skin are also induced by skin DCs to express gut homing receptors [103]. Skin barrier defects due to mutations of filaggrin, an essential protein for the skin barrier integrity, predispose to the onset of FA. Barrier function deficits can, therefore, facilitate allergic sensitization to food antigens [82,83].

## 7. Beyond Immune Cells

It is now acknowledged that both innate and adaptive immune cells and epithelial cells cooperate for the maintenance of the immunological homeostasis at the mucosal barriers. In this complex signaling network, the role of the microbiota in establishing and maintaining food tolerance is increasingly emerging.

Epithelial cells, which constitute a first defense barrier against the external environment, play a central role in allergic sensitization and immunological responses to environmental agents. Damaged skin epithelial cells release inflammatory cytokines, such as TSLP, IL-25, and IL-33, that induce cutaneous DCs and other cell types to shift immune responses from tolerance to hypersensitivity. Furthermore, the cytokines derived from tissues, in particular IL-33, also intervene in determining allergic sensitization and in triggering reactions, through the activation of ILC2, which in turn produce IL-4 and IL-13 [2]. IL-25, IL-33, and TSLP, derived by damaged epitheliocytes, function as alarmins and are involved in sensitization and maintenance of allergic reactions. These cytokines facilitate the onset and development of allergic responses to foods and are, therefore, becoming targets to treat in the management of FA [2]. In particular, IL-33 is an alarmin that also acts as a Th2 cytokine, promoting allergic reactions [104,105]. The Th2 cytokine-driven inflammation compromises the integrity of the epithelial junctions and induces specific epigenetic modifications of the epitheliocytes. Several other signals are capable of initiating allergic sensitization, including bacterial toxins and the same food components that can influence innate immunity cells, such as NKT cells. For example, the allergenicity of peanuts is enhanced by their lipidic components that are able to inhibit IL-10 production and induce inflammatory responses, facilitating allergen transfer and consequent sensitization. Furthermore, the intestinal and cutaneous microbiomes influence the functions of the respective epithelial barriers.

The intestinal microbiota variously influences the delicate balance between immunological tolerance and allergic sensitizationin the gut [83].

The microbiota is central to maintaining a state of tolerance towards food antigens [10]. The immune system can be shaped and remodeled by both antigens and microorganisms [106,107]. Through the initial encounter with microbes, the immune system learns to mount long-lasting, balanced responses. The microorganisms that colonize the intestine and the skin impact on the maturation of the immune system and influence tolerance to food [108]. Environmental factors, diet, and drugs, including antibiotics and H2 receptor antagonists, can increase the risk of FA by inducing dysbiosis. On the contrary, healthy infants likely harbor protective microbes in the gut [84]. FA susceptibility is influenced by both commensal microbiota and their metabolites. Therefore, through microbiota composition, environmental factors influence the FA risk.

Components of bacteria from the maternal intestine are already in the placenta and early colonization of the newborn’s intestine also occurs during vaginal delivery [109]. Babies born by cesarean section and those born through vaginal delivery show differences in immune responsiveness. Environmental conditions, number of family members, and coexistence with pets are among the multiple factors responsible for peculiar bacterial exposures [93]. Bacterial colonization is also variously influenced by the type of diet. For example, there are differences in the composition of the microbiota between breastfed and non-breastfed babies. Changes in the composition of the intestinal and skin microbiota are associated with FA and a protective effect of probiotics has been demonstrated [110,111]. Clostridia species from the human gut microbiota attenuate allergic responses and inhibit FA development by promoting Treg cell generation or, alternatively, by inhibiting the systemic absorption of major food allergens [110]. B regulatory cells Breg promotion and tolerance induction by Clostridium butyricum in association with immunotherapy have also been demonstrated [111,112,113,114]. The gut microbiota composition can drive the development of either resistance or susceptibility to FA in the host. Allergic infants exhibit a gut microbial community dominated by Firmicutes phylum, Ruminococcaceae, and Lachnospiraceae. The predominance of the Clostridiales species Anaerostipescaccae is associated to the expression of regulatory genes in the gut epithelium. Therefore, the microbiota, mainly Clostridiastrains, exert protective effects against FA through the induction of antigen-experienced Foxp3+ Treg lymphocytes [112]. However, Treg lymphocytes are highly heterogenous and each cell subset differs in its regulatory activity. For example, Treg cells expressing IL-4 and GATA-3 are Th2-reprogrammed cells with reduced suppressive activity which are increased in FA. On the contrary, Foxp31 Treg lymphocytes expressing the transcription factor retinoic acid-related orphan receptor gamma-t (RORgamma-t)1, which are induced by vitamin A and influenced by the intestinal microbiota, effectively inhibit Th2-skewed immune reactions underlying FA. This cell population, therefore, plays a critical role in maintaining tolerance to foods [115]. Bacteroidales and Clostridiales strains inhibit FA by inducing RORgamma-t1 Treg lymphocytes, which enhance IgA and suppress both GATA-31 Treg cells and IgE. The immune-modulating and anti-allergic effects of gut microbiota is also mediated by metabolites generated from their dietary fiber fermentation, including short chain fatty acids (SCFAs), which regulate the pool of gut Treg cells. In addition to gut microbiome, also the skin microbiome may modulate FA susceptibility: Staphylococcus aureus skin colonization increases the risk of developingsensitization to various food allergens in childhood [82].

**Figure 1:**
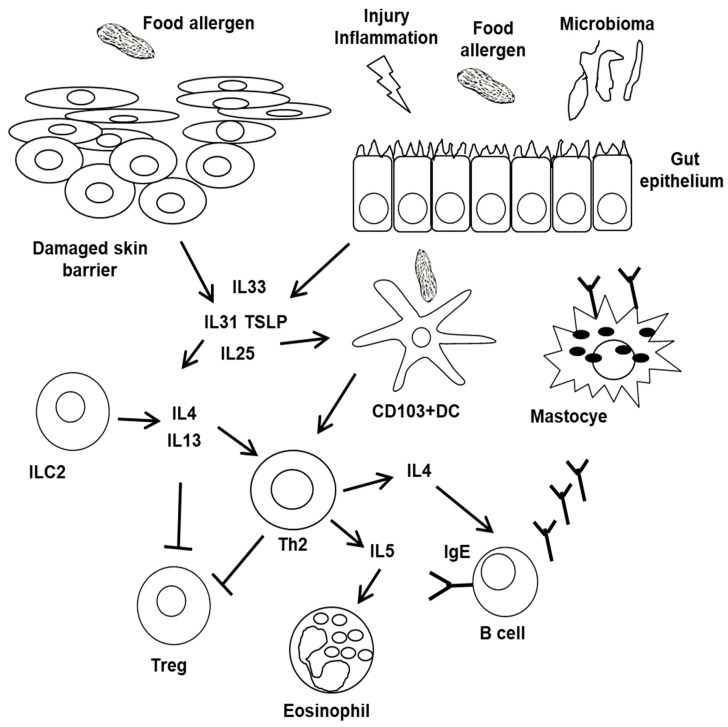
Main mechanisms by which the breakdown of tolerance to food antigens can occur. A disrupted skin barrier can provide a pathway for the breakdown of tolerance and, through the pro-inflammatory action of epithelial cells, mastcells, and innate type 2 lymphoid cells (ILC2), in addition to cytokines, such as IL-33, promotes the sensitization to food allergens. Cytokines from damaged epitheliocytes and Th2-oriented dendritic cells shift lymphocyte differentiation towards a Th2 phenotype and inhibit T regulatory cells (Treg). Th2 cytokines drive the isotypic switching of B lymphocytes, leading to the production of allergen-specific IgE that anchor to the surface of mastcells and basophils and also induce eosinophil differentiation and homing.

Figure 1 illustrates the main mechanisms by which the breakdown of tolerance to food antigens can occur.

## 8. Current Hypotheses of Food Allergy Development: Environmental Impact and Gut Microbiota

Several hypotheses, variously integrated among them, have been formulated to explain the pathogenesis of FA. Since vitamin D has well-recognized immunoregulatory and tolerogenic functions, its deficiency has been considered among the possible risk factors for FA development [40]. Even the dietary habits of Western countries and low fruit and vegetable intakes would favor allergic sensitization. The low exposure to microorganisms and the decrease in infections in early childhood would constitute a determining risk factor for the development of allergies through an imbalance of the immune responses in favor of the Th2 lymphocyte profile rather than Th1 (hygiene hypothesis). Environmental exposure to food allergens in early childhood through an altered skin barrier, in the absence of early oral food intake, by allowing skin exposure in the absence of the tolerogenic signals delivered from the gut following food ingestion, would favor allergic rather than tolerogenic responses [20]. Since tolerance to food epitope is usually established early in the life, a critical period ranging from intrauterine development to the first two years of life (the first 1000 days) has been identified. During this period, also knownas “window of opportunity”, the individual’s susceptibility to develop allergies is established [20,116]. Treg environment at the maternal–fetal interface is protective for the fetus and maternal factors contribute to induce tolerance to allergens in neonates [117,118]. Awareness of the lasting effects of environmental factors on the immune system in this very early period of life has profoundly conditioned the prevention strategies for FA. In the past it was recommended to avoid as long as possible the introduction of potentially allergenic foods into the diet of children at greater risk of allergies [119,120,121], butin recent years most of the guidelines in this regard have been radically changed [3,122,123,124,125]. The new dietary recommendations and practical guidelines for the prevention of FA have, therefore, been updated based on the results of recent studies [126,127] demonstrating that the practice of excluding food allergens from children’s diet has contributed to the significant FA increase of the last years. On the contrary, regular consumption of food antigens since early childhood elicits protective sustained immune responses. Accordingly, a sustained consumption of major allergenic foods, such as peanuts, is now recommended from the first months of life. In particular, based on the results of LEAP and EAT trials, a reduction in the risk of peanut allergy could be obtained if peanut is introduced between the ages of 4 and 11 months. In addition, current FA guidelines advocate early introduction of peanut between the ages of 4 to 6 months to infants who are considered at high risk of developing a peanut allergy, introduction of peanut-containing foods to children with mild to moderate eczema around 6 months of age and to children with no eczema or food allergy freely into the diet, in accordance with family preferences and cultural practices [6].There are currently no precise guidelines for early introduction of egg and milk in the diet of infants at risk, although egg introduction at 4 to 6 months and cow’s milk within the first 14 days of life are suggested [128]. The early introduction of food allergens into the diet could, therefore, representa promising prevention strategy [129,130,131,132,133,134]. Furthermore, the maternal consumption of common food allergens (mainly milk and peanuts) during pregnancy and a greater food diversity in childhood diet could be further useful tools for reducing allergic risk [117,118,135].

Finally, the currently most investigated hypothesis is that the altered microbiota compositionis a risk factor of FA. In particular, it has been suggested that some bacterial strains, as well as microbial diversity, support Treg lymphocyte maturation, favoring tolerance to food antigens. On the contrary, the indiscriminate use of antibiotics could facilitate the onset of allergies by destroying the microbiota diversity [95,110]. In addition to the presence of specific bacterial strains, also dietary substrates and their metabolites, such as short chain fatty acids, could influence the development of food allergic sensitizations. It has been suggested that diets rich in fiber may influence the balance between the different commensal bacterial strains in the gut microbiota [115,136].

## 9. Novel Treatment Strategies

Based on the recent discoveries on the immunological mechanisms underlying FA and the biotechnological progress in personalized diagnostic and prognostic evaluation, the therapeutic approach is substantially changing. As a result, new management of FA is taking shape by the achievement of safe and effective therapies against key molecular targets and pathogenetically relevant signal pathways [137,138].

### 9.1. Specific Immunotherapy

Currently, specific immunotherapy strategies to desensitize FA patients, are proposed (FAIT) [139,140]. Food allergy immunotherapy (FAIT) is aimed to achieve a permanent unresponsiveness to food allergens or at least to increase the threshold dose of food necessary to trigger an allergic reaction. Overall, specific immunotherapy, therefore, aims to guarantee a certain degree of protection from accidental exposure and to improve the quality of life [2,141,142]. FAIT induces several immune changes, including the shift of allergen-specific Th2 lymphocytes towards IL-10 and TGF-β-producing Foxp3+ Treg cells and the increase of regulatory B-cells (Breg) that secrete IL-10, with reduced responses upon allergen trigger and downregulation of Th2 inflammation. The decreased reactivity of effector cells of anaphylaxis and the downregulation of their release ability and degranulationare among the early modifications induced by FAIT. Specific IgE levels are decreased by FAIT, whereas allergen-specific IgG4 are increased, competing with IgE to dampen the allergic response [143,144]. In addition, FAIT also induces anergy and functional exhaustion of other cells, probably resulting in their elimination.

Different methods for FAIT have been proposed [145,146], including oral (OIT), sublingual (SLIT), and epicutaneous (EPIT) routes. OIT involves the ingestion of low and progressively increasing daily doses of allergen. A liquid allergen extract is administered under the tongue in SLIT. In the EPIT, the administration route is the skin surface through special adhesive devices containing the allergen [147]. Although associated with the greatest number of adverse events (mostly gastrointestinal disorders), OIT seems to be the most effective immunotherapy at the moment. The administration of the initial doses under clinical control is recommended [20]. Some patients who perform OIT may also develop eosinophilic esophagitis for which desensitizing treatment is stopped [148,149]. The OIT rationale and its greater efficacy compared to other forms of specific immunotherapy in FA is based on the fact that the ingestion of a food antigen preferentially determines a tolerogenic active immune response rather than allergic sensitization [150]. Currently, the OIT is recommended in cases of persistent allergy to peanuts, cow’s milk, and chicken eggs in allergic children from 4 to 5 years of age, in which it has been shown to be able, at least during therapy, to increase the threshold of clinical reactions [151].

Modified foods with reduced allergenicity may allow for immune modulation with less risk of allergic reaction. Baked milk and egg diets are increasingly used in the management of milk and egg allergy, rather than avoidance. Cow’s milk and hen’s egg IgE-dependent allergies are among the most common FA in children. However, the majority of children allergic to egg or milk in its raw form are able to tolerate egg and milk in baked goods. Extensive heating of milk and eggs induces changes in the conformational structure of the epitopes, effectively destroying them, thus reducing their allergenicity. There are evidences that baked foods might have a positive effect on the acceleration of allergy resolution in children with cow’s milk and egg IgE-dependent. However, it is unclear whether children who develop tolerance do so because they have ingested low levels of egg or milk in baked products or their better prognosis is simply an indicator of a phenotype that is less likely to be persistent. Children who tolerate baked milk and egg may have smaller skin prick test, lower specific IgE, less basophil reactivity, and more peripheral T regulatory cells [152]. Recently, a randomized controlled trial of baked milk ingestion did show a significant change in ability to eat unheated milk at the end of 1 year [153]. A high-dose rush OIT protocol in which egg-allergic children had a 5-day dose buildup followed by 5 months of eating an undercooked egg has been well tolerated and efficacious [2,154].

### 9.2. Pharmacological Treatments

Adrenaline, administered early at the first clinical signs of anaphylaxis after ingestion of the culprit food, is crucial to prevent the fatal outcome of anaphylactic reactions, reversing, in a few minutes, hypotension, shock, and other allergic symptoms (urticaria, bronchospasm, edema, gastrointestinal manifestations, and so on) [155]. It is, therefore, a quite specific medication as a rescue tool to be used in particular for those patients who have been prescribed to take it. In the case of itching and hives, antihistamines blocking specific H1 receptors could also be useful, whereas H2receptor blockers could be used to treat gastrointestinal symptoms [156].

Unlike allergen-specific immunotherapy, which requires the administration of the culprit food, new nonspecific therapeutic approaches targeting at the upstream events that regulate the function of both inducer and effector cells involved in FA, as well as at the IgE production, are independent of the allergen and aim to suppress the native and adaptive responses that drive the allergic reactions [2]. Personalized pharmacological and nutritional interventions [157], targeted therapies with biologics [158], including the monoclonal anti-IgE antibody omalizumab, dupilumab (anti-interleukin-4Ra), anti-IL-5 monoclonal antibodies (reslizumab and mepolizumab), and finally the reconstitution of a microbiome composition capable of inducing tolerance, for example, through Lactobacillus bifidus and Clostridium fragilis administration or through fecal transplantation [159,160,161], are innovative therapeutic tools that are becoming part of the modern FA treatment strategies.

Omalizumab, by linking the Fc region of IgE antibodies and blocking the binding of IgE to FcεRI on mastcells and basophils, prevents the degranulation of these cells and the consequent clinical manifestations triggered by the released mediators [29,30,162]. Omalizumab, already successfully tested in various IgE-mediated pathologies [28], is also usefully associated with the OIT to treat FA. Before starting OIT, to obtain a reduction of the levels of free IgE and to induce a downregulation of the FcεRI expression on the surface of mastcells and basophils, patients are treated with biweekly or monthly omalizumab injections for 8 weeks [163]. Thereafter, both therapies are administered in association for an overlap period, until biological drug suspension [164]. Other biologics targeting key pathogenetic cytokines are also promising in FA. Among them, monoclonal antibodies against IL-5, namely reslizumab and mepolizumab, have been successfully administered in the treatment of eosinophilic esophagitis [165]. These monoclonal antibodies are, therefore, useful for preventing and treating unwanted reactions triggered by specific immunotherapies. Dupilumab, recently approved for atopic dermatitis, has also been tested in peanut allergy and in association with OIT. It suppresses IL-4 and IL-13 and, as a consequence, the IgE class-switching and the activation and expansion of effector cells, thus abolishing food allergic responses. Etokimab, an anti-IL-33 antibody, has been successfully tested in adult patients affected by peanut allergy [2]. Designed ankyrin repeat proteins (DARPins), able to remove IgE from FcεRI binding, as well as TSLP and IL-25 targeting, may potentially represent a new alternative approach [138]. Treatment strategies with the goal of modifying the gut microbiota are also in development. Fecal microbiota transplantation (FMT) performed from wasper formed from healthy to allergic subjects could be a promising therapeutic strategy. Alternatively, cocktails of protective bacterial strains, including Clostridiales or Bacteroidales strains, such as B fragilis, could also be considered in protection from FA. Probiotic supplementation with Lactobacillus rhamnosus GG has been utilized during OIT for peanut allergy, and prebiotics, including oligofructose, acidic oligosaccharides, and long-chain inulin, in combination with the prebiotic Bifidobacterium breve M-16V, have also been administered in clinical trials for FA with some success [165,166,167,168,169,170,171,172,173].

## 10. Concluding Remarks

Tolerance is the state of healthy nonreactivity of the immune system to common and harmless food antigens. Desensitization, on the other hand, consists of a temporary raising of the allergic reactivity threshold and is supported by immunological mechanisms other than those involved in the tolerance, which is the basis of permanent anallergic immune condition. Immunotherapy often causes a state of desensitization rather than prolonged or permanent nonresponsiveness.

Thanks to the growing understanding of the immunological mechanisms underlying desensitization to food antigens, new therapeutic strategies targeted at important immunological checkpoints for the management of FA are in progress, and will soon arrive in clinical practice. Diet, probiotics and prebiotic supplementation, and FMT are potential therapeutics for FA treatment and prevention. Future FA prevention strategies could be aimed at limiting the interruption of the skin barrier, colonizing the intestine with tolerogenic commensal bacteria, and introducing allergenic foods early in the diet. Therapeutic immunological targets of FA could be both T-specific effector cells for food allergens and the secreting IgE plasma cells. The expansion of allergen-specific Treg cells is probably the most promising therapeutic strategy. However, despite the seen enormous advances and the availability of innovative diagnostic and therapeutic tools, the management of food allergy is still an evolving field of research.
